# Using the Evidence-Development-Validation-Consensus (EDVC) Approach to Develop and Validate maxSIMdrone: A Training Program for Healthcare Professionals to Provide Cardiac Arrest Care Using Drones

**DOI:** 10.7759/cureus.40729

**Published:** 2023-06-21

**Authors:** Bruno Gino, Andy Benson, Adam Dubrowski

**Affiliations:** 1 Emergency Medicine, Memorial University of Newfoundland, St. John's, CAN; 2 Health Sciences, Ontario Tech University, Oshawa, CAN; 3 Central East Prehospital Care Program, Lakeridge Health Hospital, Oshawa, CAN

**Keywords:** drones, emergency medicine physician, out of hospital cardiac arrest, simulation trainer, simulation medicine

## Abstract

Introduction

The challenges of delivering cardiac arrest (CA) courses in rural and remote (R&R) locations worldwide have been further exacerbated by the COVID-19 pandemic. However, it is important to note that this problem has always existed. The implementation of social distancing measures to combat the pandemic has had a significant impact on healthcare and medical education, particularly in relation to the training of students, laypeople (LP), and healthcare professionals (HCPs) in CA care. The combination of pandemic restrictions and pre-existing difficulties faced in R&R locations and large cities has disrupted the provision of comprehensive medical education. The suspension of basic life support and defibrillation (BLSD) courses during the pandemic may have negatively affected pre-hospital care for CA. However, it is essential to acknowledge that challenges in delivering these courses in R&R areas predate the pandemic.

Materials and methods

A 2021 epidemiological study in the Brazilian Amazon identified CA as the primary cause of death, followed by COVID-19. This highlights the importance of providing BLSD courses and training to emergency medical service (EMS) personnel in R&R locations. Even during a pandemic. Researchers from Ontario Tech University and Memorial University School of Medicine developed a drone with a simulation scenario to train HCPs in automated external defibrillators (AED) operation and guide LP in safe use through BLSD protocols. A literature review showed that different training methods yielded similar outcomes. Based on these findings, the evidence-development-validation-consensus (EDVC) hybrid approach was used to develop and validate an online training program using a learning management system (LMS) as a model.

Results

Teaching HCPs and LP in R&R locations, such as northern Canada and the Brazilian Amazon, presents challenges due to limited resources and internet access. One potential solution lies in the utilization of remote online LMS that facilitate the administration, documentation, tracking, reporting, automation, and delivery of educational courses and training programs. The literature review indicated that mixed training approaches, including face-to-face, online, and hybrid formats, produced similar outcomes in learning assessment, self-confidence, performance, skills, and knowledge acquisition. These findings support the viability of using LMS as a model to develop and validate a course where drones deliver AEDs and provide training to HCPs and LP in R&R locations. A comprehensive training program should encompass cognitive, affective, and psychomotor learning domains, addressing various skills and knowledge aspects.

Conclusion

This research study develops and validates LMS teaching methods to support a training program for HCPs and LP in using AEDs delivered by drones. The program combines design-based research and consensus development methods, such as design thinking and think-aloud observations. Drones are used to provide AEDs and develop simulation scenarios for training in R&R locations. The hybrid approach ensures a valid and evidence-based training program. The study presents the EDVC approach used to enhance the maxSIMdrone training program, enabling effective out-of-hospital CA care. The program incorporates participant feedback and improves knowledge and techniques in AED use. It has the potential to improve patient outcomes in resource-limited R&R locations.

## Introduction

The COVID-19 pandemic led to substantial changes in healthcare and medical education, with social distancing measures implemented to prevent the spread of the SARS-CoV-2 virus [1]. This had a profound effect on medical education, especially for students, laypeople (LP), and healthcare professionals (HCPs) without prior training in cardiac arrest (CA) care. Due to restrictions [2], they were unable to access simulation labs and classrooms in educational institutions. However, these restrictions had varying effects on medical education in rural and remote (R&R) locations and large cities [3]. For instance, certain studies indicate that public basic life support (BLS) courses at training centers were entirely suspended during the pandemic peak, which may have had detrimental consequences for pre-hospital care in R&R locations, as CA remains the leading cause of death worldwide, claiming approximately 27.9 million lives annually and accounting for 31% of global deaths [4-5].

In the Brazilian Amazon, a descriptive and quantitative epidemiological study has revealed that CA is the leading cause of death (15.05%), followed by COVID-19 (10.29%) in 2020 and 2021 [6]. These statistics highlight the importance of providing emergency medical service (EMS) personnel in R&R locations with courses and training in BLS and guidance on how to instruct LP to perform cardiopulmonary resuscitation (CPR) techniques through mobile communication tools, even during a pandemic.

In CA specifically, medical literature strongly advocates for reducing the time to initiate the use of automated external defibrillators (AED) during CA, as every minute delay in defibrillation can increase mortality by 7-10% [7]. Therefore, it is crucial to reduce the time a victim spends without defibrillation to enhance survival rates. Recently, in 2022, researchers from the Ontario Tech University (OTU) and the Faculty of Medicine at Memorial University (MUN) in Canada developed a drone equipped with a simulation scenario to teach HCPs how to operate AEDs and to guide LP to use them safely and effectively through basic life support and defibrillation (BLSD) protocols [8]. Nonetheless, before implementing AED delivery systems by drones, it is imperative to train HCPs and LP in these R&R places who will send or receive the devices on their effective and safe usage based on evidence.

However, teaching HCPs and LP in R&R locations poses significant challenges, particularly in areas such as the north of Canada and the Brazilian Amazon, where HCPs require continuous training and work in remote areas with limited resources, power, and internet access [8]. The Brazilian Amazon presents a unique challenge, as HCPs in the heart of the forest have limited access to resources [6]. One potential solution is the use of a remote online learning management system (LMS), which provides administration, documentation, tracking, reporting, automation, and delivery of educational courses and training programs [9]. However, given the lack of resources and unstable internet in R&R locations like the Amazon, an LMS that also works offline for skill acquisition would be necessary to effectively train HCPs and LP on the use of AEDs delivered by drones.

For this purpose, the researchers conducted a literature review that revealed that mixed, face-to-face, and online training for AED use all yielded similar outcomes in terms of learning assessment, including self-confidence, performance, skills, and knowledge acquisition for individuals with little or no prior experience with AEDs [10]. Based on these findings, it is reasonable to suggest that teaching HCPs and LP in R&R locations to use AEDs delivered by drones is a viable option. Also, the LMS is a crucial tool that provides remote teaching, with flexible availability and feedback [9]. Due to this reason, a comprehensive EMS training program should cover various aspects including cognitive, affective, and psychomotor learning domains. It should focus on developing skills and knowledge in comprehension, analysis, synthesis, and evaluation. Additionally, it should address emotions, attitudes, physical skills, and coordination, involving perception, mechanism, complex open response, adaptation, and origination [8-10].

This research study focuses on utilizing LMS teaching methods as a model to support a training program for HCPs and LP in the usage of AEDs delivered by drones. The aim is to provide training specifically for individuals with little or no prior knowledge of these devices. The study follows the initial phases of the design-based research approach, which involves communication among clinical educators, AED specialists, and EMS professionals working in R&R locations [11]. This collaboration aims to create a successful program design that effectively trains participants in using AEDs delivered by drones. To encourage innovation and creativity in this process, consensus development methods will be applied in this research as this approach is favored by designers [12].

In the context of education, the availability of AEDs and the implementation of BLSD courses have contributed to standardizing and organizing medical care for CA [10]. It has been observed that the survival rate of CA patients significantly improves when defibrillation is performed within five minutes of the onset of the condition [13]. Therefore, it is crucial to provide proper training for HCPs and LP to ensure they can effectively use AEDs delivered by drones, leading to better patient outcomes and response time especially by enabling immediate recognition of CA, facilitating audio and visual contact with the emergency system, initiating high-quality CPR, and utilizing on-site AEDs as soon as they become available [8].

However, in order to develop a training program that is both valid and evidence-based, the authors propose a hybrid approach that combines elements of evidence generation and consensus development methods [14]. By integrating evidence-based practices with innovative consensus development methods, the goal is to create a comprehensive training program that effectively equips participants with the necessary knowledge and skills to utilize AEDs delivered by drones in emergency situations.

## Materials and methods

The evidence-development-validation-consensus (EDVC) approach

The local Lakeridge Health Research Ethics Board granted an exemption and issued approval (approval no.: 2023-003) for this research [15].

The EDVC is a comprehensive approach created specifically for this study to address the need for collaboration among clinicians, educators, and engineers without creating expertise silos, that combines a technical report with a simulation scenario, a literature review, and the use of the modified design thinking (MDT) process and think-aloud observation (TAO) methodology [8,10,12,16,17].

The first step in this approach was to develop a technical report with a simulation scenario and next to conduct a systematic review to identify publications about learning assessments on the use of AED and CPR protocols [8,10]. These components were published separately, contributing to the comprehensive nature of the EDVC approach.

In the second step of the approach, the MDT process was employed to generate innovative ideas, followed by the utilization of a TAO method to consolidate and validate the new content derived from these ideas [12,16]. This approach was crucial for the current research as it allowed for the inclusion of additional perspectives and insights into the training program's development and validation. 

**Figure 1:**
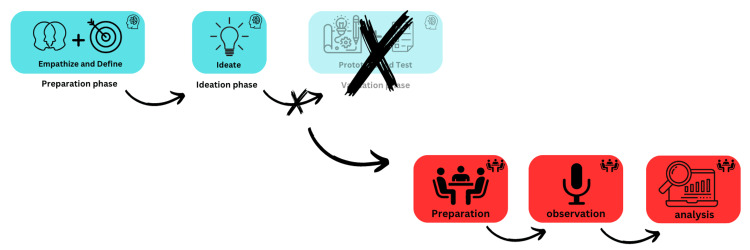
The MDT and TAO Method Together. MDT: modified design thinking TAO: think-aloud observation This image was created by the authors using Canva, a platform that enables the publication of their own images.

**Figure 2:**
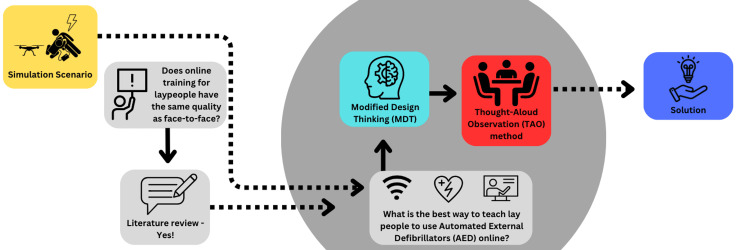
The EDVC Approach. MDT: modified design thinking TAO: think-aloud observation AED: automated external defibrillators EDVC: evidence-development-validation-consensus This image was created by the authors using Canva, a platform that enables the publication of their own images.

The MDT process involved interactive rounds of brainstorming and collaboration with a group of experts proficient in using AEDs during CA incidents. However, the authors replaced the prototype and testing phases from the MDT process with the TAO method to allow AED experts to provide feedback that is acceptable for the training program [12,16]. These experts played a significant role in the creation and validation of a training program specifically designed for the LMS mode, where drones are employed for the delivery of AEDs. The TAO method was an integral part of this process, allowing for the observation and assessment of the experts' thought processes and interactions with the training program's concepts and content. The MDT and TAO methods together and the EDVC approach respectively are available below (Figure 1 and Figure 2).

Experts

For the MDT process, four participants volunteered and received pseudonyms: a medical doctor (Alpha, Brazilian Amazon), two nurses (Beta and Gamma, Brazilian Amazon), and a nursing technician (Delta, Brazilian Amazon). Three had experience with BLSD and one of them, who was a nursing technician, had little or no knowledge of the use of AEDs.

For the TAO methodology, three participants volunteered and received pseudonyms: a medical doctor (Participant A, Brazilian Amazon), a nurse (Participant B, Brazilian Amazon), and a paramedic (Participant C, Lakeridge Health Hospital in Canada). All three had extensive experience with BLSD.

The maxSIMdrone

The training program developed for this research, aimed at training HCPs and LP on operating AEDs delivered by drones in R&R locations, is referred to as the "maxSIMdrone program." The maxSIMdrone program utilizes BLSD stages in the lifesaving process and an evidence-based model for structuring the online and remote course called LMS to facilitate the acquisition of psychomotor skills [8-9]. In BLSD, the "stages in the lifesaving process" refers to a sequence of critical steps that, when followed in a timely manner, can greatly improve the chances of survival for a person experiencing CA [8]. An LMS is a software application used for the administration, documentation, tracking, reporting, automation, and delivery of educational courses and training programs [9]. It is important to note that the maxSIMdrone program introduces several new components, including the drone itself, the LMS with offline components, and the content being delivered. To enhance clarity and understanding, operational definitions and explanations of these elements will be provided to ensure the reader comprehends their roles and significance within the context of the study.

**Figure 3:**
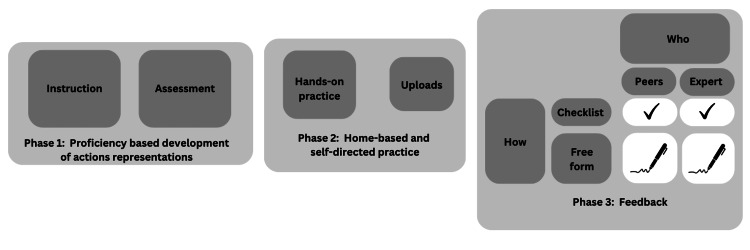
Model Illustrating Three Phases of Learning and the Components Required for the LMS. LMS: learning management system This image was created by the authors using Canva, a platform that enables the publication of their own images.

The activities in the online LMS are organized in three distinct phases (Figure 3):

In Phase 1, learners receive active instructions and assessments, such as instructional videos that demonstrate different techniques, errors, and alternative approaches. They review these materials and use assessment rubrics to identify errors and alternatives [9]. However, before progressing to Phase 2, learners must meet proficiency criteria established by the course manager. This involves the course manager identifying a predetermined number of errors or alternative approaches. Once these criteria are met, learners can proceed to the next phase, where they can practice using simulators and specialized equipment.

In Phase 2, learners have the opportunity to enhance their skills through hands-on practice using simulators and training equipment. These simulators and equipment, such as the PHOENIX drone, can be made available through 3D printing technology [8]. Institutions of education or even the government can acquire these resources if it aligns with their interests. Here, they practice using simulators designed specifically for this purpose. They can refer back to the instructional materials in Phase 1 for further guidance, and once satisfied with their performance, record a test attempt and upload it to the LMS for feedback [9].

In Phase 3, learners have the opportunity to receive feedback on their test from their peers, an expert, or a combination of both. Feedback can be based on checklists or a free form [8-9]. Using peers for evaluation further expands learning opportunities, as participants providing feedback engage in observational practice and error detection [9]. 

**Figure 4:**
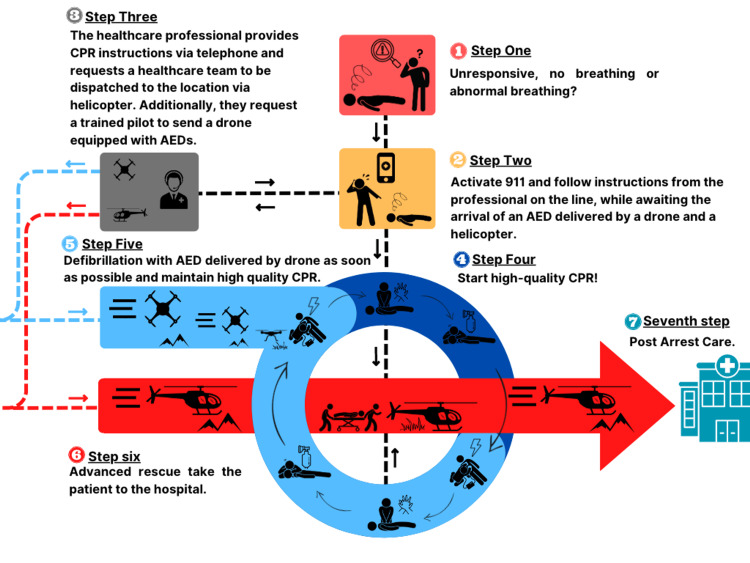
The Stages in the Lifesaving Process. AED: automated external defibrillator CPR: cardiopulmonary resuscitation 911: emergency services This image was created by the authors using Canva, a platform that enables the publication of their own images.

One important component that could be implemented for maxSIMdrone is the ability to practice hands-on training. The tools that could be used for this are the PHOENIX drone, an AED, and a BLSD training simulation scenario already developed to teach how to operate AEDs delivered by drones [8]. The stages in the lifesaving process can be seen below (Figure 4).

Conducting the EDVC approach

As part of the EDVC approach, the MDT process and the TAO methodology applied for this study will be discussed below in more detail.

Modified design thinking (MDT) process

Participants

The MDT process was conducted through a two-hour video conference using Google Meet (Google Inc., Mountain View, California, USA) and involved all four participants, a Brazilian doctor (Alpha), two Brazilian nurses (Beta and Gamma), and a Brazilian nurse technician (Delta). The purpose of this process was to develop a solution to the problems encountered in R&R locations by empathizing with the researchers who developed PHOENIX and the fundamental aspects of BLSD, defining the problems clearly, and generating ideas to meet the identified needs and solving the problems [8,12].

Procedure

Empathy stage: During the first phase of the process, called empathy, participants were provided with a prototype of the maxSIMdrones (v1.0) based on the previous study [8]. The experts were then asked to put themselves in the shoes of the researchers who developed PHOENIX and the fundamental aspects of BLSD to gain a deeper understanding of the issues faced in R&R locations. They were encouraged to share their thoughts and experiences on the challenges encountered in these locations.

Defining the problem stage: After the empathy phase, the participants moved on to clearly defining the problems encountered in R&R locations. This was done through a brainstorming session where participants identified and discussed the issues they had encountered in these locations [12,18]. The problems identified were then organized and categorized based on their relevance to the project.

Generating ideas stage: In the final stage of the MDT process, the goal was to create ideas that would meet the needs and solve the problems that were identified [12,18]. Experts were encouraged to think creatively and come up with new and innovative ideas. These ideas were written down by hand and recorded by researchers. Then, they were incorporated into the latest version of the maxSIMdrones prototype (v2.0).

Data Analysis

During the data analysis phase, the use of a prototype and notes proved instrumental in capturing and visualizing the ideas generated throughout the process. As shown in Appendix A, the prototype of maxSIMdrones (v1.0) was developed during the MDT process, accompanied by notes from researchers and comments from the experts. Subsequently, a modified version (v2.0) of the prototype was created based on the feedback received [17]. This iterative MDT process, which formed part of the data analysis, was a collaborative effort involving experts from various healthcare professions from R&R places. It commenced with empathy, followed by a clear definition of the challenges faced in R&R locations. Finally, innovative ideas were then generated to effectively address the identified needs and resolve the problems at hand.

Think-aloud observation (TAO) methodology

The TAO methodology was selected as an appropriate approach for the next stage of this study. As per previous research, it has been demonstrated that using TAO to gain qualitative insights is a valid and effective approach, especially when the focus is on understanding participants' experiences [16]. Moreover, it has been established that a smaller number of participants is generally considered adequate for qualitative studies, including those using TAO [19].

Participants

In line with this, the authors decided to utilize TAO with three specialists involved in prehospital care, including a Brazilian doctor (Participant A), a Brazilian nurse (Participant B), and a Canadian paramedic (Participant C). These participants were selected based on their expertise and knowledge of the subject matter. It is important to note that using three participants in TAO is a widely accepted and appropriate approach for gaining valuable insights and improving programs, as supported by previous studies [19-20].

Procedure

The TAO methodology involves a three-step process, including determining the objectives, piloting the process, and executing the TAOs [16]. By employing this approach, the researchers are confident that they will obtain valuable insights into the prototype of the maxSIMsaves (v2.0) training program and make necessary improvements for a final version.

"Determine" Stage

In the "determine" stage, based on the ideas of participants in the MDT process, the authors established the foundational elements for the next stage in the TAO methodology [16]. The authors carefully identified what aspects of the program they wanted to evaluate and which specific participants they should recruit to ensure their relevant expertise and knowledge would be applicable to the research [16,19]. The authors also created tasks to test the usability of the course elements, which they would use in the pilot and "do" stages [16]. After careful consideration, it has been decided that only professionals who have had experience with AEDs and BLSD will be chosen to carry out the analysis of our training program using the TAO methodology. This decision was made based on the fact that these professionals have the necessary expertise and knowledge to generate valuable and high-quality information for our study [16,19-20]. By selecting individuals who have experience with AEDs, the authors can ensure that the feedback they receive from the TAOs is accurate and insightful. Their expertise will allow them to provide a unique perspective on the training program, identifying strengths and weaknesses that might not be apparent to those without such experience. This approach will not only enhance the quality of our study but will also provide us with a better understanding of how our training program is being received by those who have used AEDs in the past. The authors believe that this decision will ultimately result in a more comprehensive and informative analysis of our program.

Pilot Stage

During the pilot stage, the authors rehearsed, tested, and established realistic timing estimates for the TAO process [16]. Additionally, the authors conducted a thorough review of the training program content on maxSIMdrone (v2.0) with a team of experts from this research study, a physician, and a paramedic. This review was vital in ensuring that the program content was accurate, relevant, and up-to-date. Through this pilot stage, the authors were able to validate the data and the wording of the tasks to be used in the TAOs. The authors also addressed any issues that arose during the pilot stage to ensure that they were fully prepared for the actual user experience (UX) testing with recruited participants in the "do" stage [16].

"Do" Stage

Finally, in the "do" stage, the authors conducted the actual UX testing with the three recruited experts. The researchers used the thinking aloud technique to capture preference and opinions data simultaneously [20-22]. This technique requires participants to verbalize their thoughts and reactions as they navigate through the training program and tasks [21-22]. By doing so, the authors were able to gain insights into the experts' emotions, expectations, and preconceptions about the program.

During the TAO sessions, the researchers asked the participants to complete the tasks they had created beforehand [16]. As they worked through the tasks, they were asked to describe what they were thinking and feeling in real time. We also encouraged them to ask questions and provide feedback as they went through the program. The authors made sure to remain neutral and non-invasive during the sessions, only prompting the participants when they were struggling or had been silent for too long [21-22]. Researchers also made sure to take detailed notes and record audio of the sessions to analyze later.

Exit Interview

After completing the tasks, the researchers conducted an exit interview with each participant. The authors asked them to provide feedback on the training program, including their overall impression, strengths and weaknesses, and suggestions for improvement [16,21].

Debrief

After the post-test session, researchers debriefed the participants, thanking them for their time and providing them with an opportunity to ask any questions or seek clarification [16]. The authors also informed them of any plans to incorporate their feedback into the training program.

After the TAO sessions were completed, collected data were analyzed. The authors reviewed the audio recordings and compared them to their notes to ensure that they had captured everything accurately. Researchers also created a list of the participants' feedback and suggestions to improve the program. Table 1 shows the tasks that participants performed in the "do" stage about maxSIMdrone (v2.0).

**Table 1:** Tasks. AED: automatic external defibrillator R&R: rural and remote LMS: learning management system

Task	Questions for Think-Aloud Observations (TAOs)
#1	1 - Phase 1: Regarding the learning objectives presented in Phase 1, please share your opinion verbally.
#2	2 - Phase 1: Please provide feedback on the instructional videos presented in Phase 1 and express your thoughts aloud. How can they sufficiently cover the necessary skills and knowledge for operating drone-delivered AEDs at R&R sites? Please share your opinion verbally.
#3	4 - Phase 1: Evaluate the training equipment provided in the course and share your thoughts aloud. Are they user-friendly and capable of simulating real-life scenarios effectively? Do they meet the requirements for training healthcare professionals and laypeople to operate drone-delivered AEDs at R&R sites? Please share your opinion verbally.
#4	5 - Phase 1: Imagine that you are preparing for the next phase of the training and consider the importance of providing voice-recorded questions. Some individuals participating in the training may have difficulty reading, so when do you think it's essential to offer questions verbally? Were the voice-recorded questions easy to comprehend and utilize? Please share your opinion verbally.
#5	6 - Phase 2: Envision yourself participating in this training. Do you believe it is crucial to include photographs in this session? Please share your opinion verbally.
#6	7 - Phase 2: Imagine that you are going to participate in this training. What is your opinion about describing what equipment will be used, that the scenario should be as simple as possible? That should emphasize the importance of being ethical with the victims' information during communication and highlight that other people not involved in the service can hear all the communication? Please share your opinion verbally.
#7	9 - Phase 3: Imagine yourself participating in this training without knowing how to read or write, but you can record videos and send them to receive feedback. What are your thoughts on this option, and how would you feel about having it? Please share your opinion verbally.
#8	Please give your feedback on the overall structure and design of the LMS and the online simulation-based training model, and express your thoughts aloud. Is the course easy to navigate? Are the instructions and materials clear and concise?

Data Analysis

The researchers analyzed the data collected during the TAO from each participant to identify common themes and patterns in the participants' feedback. We organized the feedback into three categories as 1 - usability, 2 - content, and 3 - design, and prioritized the issues based on their frequency and severity [16,21]. The authors prepared a report summarizing the findings from the TAOs, including the feedback received from each participant, common themes and patterns, and recommendations for improving the maxSIMdrone [21]. Appendix B shows the findings report.

## Results

MDT process results

The MDT process was utilized to improve the LMS-based training program, which was previously called maxSIMdrones (1.0). Version 1.0 was divided into three phases: phase 1 - proficiency-based development of action representations (instruction), phase 2 - home-based and self-directed practice (hands-on practice), and phase 3 - feedback. The participants provided their comments and feedback, which led to the creation of version 2.0 of the training program (Appendix A).

Phase 1 (Instruction)

During the first phase, which focused on instruction, the participants recommended changes to the online training model. One of the main requests was that the training program should be accessible offline as well (Alpha comments: “We have difficulty accessing the internet, so I believe the information must be offline as well"). Additionally, the participants recommended the use of a helicopter as the means of transport in the training program to simulate what happens in the real world in R&R locations (Gamma comments: "I think it's good to replace the ambulance for a helicopter because we use helicopters here”). They also suggested that the means of communication between the provider and the HCPs should be a combination of radio and cell phone with internet (Beta comments: "We use radio and cell phones here, add radios here too”, Alpha comments: “We use radios as well but sometimes WhatsApp app when we have internet access”). The instructional videos should be recorded in a remote environment to give the participants an idea of what to expect during the training (Gamma comments: “I think the video should be recorded inside the forest, so people can understand how it would be. It would be nice to see a video recorded in a remote location”). The videos should contain all the step-by-step of the BLSD stages in the lifesaving process, how to use an AED, and include all necessary equipment such as radios and cell phones (Alpha comments: "I think the video should be showing how to do CPR and use the AED and all how to use the equipment as well"). The participants also recommended the inclusion of videos and pictures of all equipment and an oral test in addition to the written test, to cater to those who have difficulty reading and writing (Alpha comments: "I believe it should have audio, some people can't read", Beta comments: "Yes, definitely should have questions in audio format"). 

Phase 2 (Hands-on Practice)

In the second phase, which focused on hands-on practice, the participants suggested adding pictures of walkie-talkies and step-by-step videos from tablets or cell phones on how to carry out the training (Gamma comments: “You could keep pictures of all equipment here as well”). The participants recommended including information on how to call for help through WhatsApp, walkie-talkie, and cell phones (Alpha comments: “Explain here how people should call for help using radio, phone, or WhatsApp"). They also suggested keeping the scenario as simple as possible and adding an audio description to make it easier to understand. The importance of preserving patient data was emphasized during training, and the training program should include this aspect to ensure participants understand its significance. Also, the participants suggested that a local resident could serve as an "information bridge" between the health professional and the person providing assistance to the victim with cardiac arrest via radios (Alpha comments: "The scenario must be the simplest and easiest. Some people have difficulty following complex scenarios”, Alpha also comments: "Put the information here that sometimes the radios of the health professional and the provider are far away and in some cases a resident who has a radio and is between the two can serve as a bridge of information between them”).

Phase 3 (Feedback)

In the feedback phase, the participants recommended adding video explanations that would illustrate where the participant went wrong and where they got it right, in addition to the written text and checklist feedback provided (Alpha comments: "Here it would also be interesting to put audio and videos, mainly video with the feedback explaining where it went wrong and how the participant can correct it", Delta comments: "Adding video explaining how to do things correctly would be very good"). 

Think-aloud observation (TAO) methodology results

The research team used the TAO methodology to obtain feedback from three specialists, a Brazilian doctor and a nurse, and a Canadian paramedic, on the prototype of the maxSIMsaves (v2.0) training program. The feedback received was analyzed to identify common themes and patterns and was divided into usability, content, and design [16]. 

Usability

Participant A suggested that the interface must be easy to use and that drawings and figures may be easier to interpret than text (“That's what I think. Interpreting a text may be more difficult than interpreting drawings and figures”). The participant also highlighted the possibility of communication failure and the need for a third person to act as a communication source (“You put the 'person bridge' here, right? If sometimes communication fails, a third person will have to play the role of communicator source”). Participant B recommended adding more training tools, specifically using a mannequin for practical training (“Thinking about training, it's still not real life, but we should also consider real events. When I learned to handle it, it was with a mannequin”). They also suggested including oral tests for indigenous populations during training (Participant A: “Yes, I think it's important to include oral tests for indigenous health professionals because some of them don't speak Portuguese”). Participant C suggested that the video should focus on critical points and important information should be highlighted using a color overlay (“Critical points where you have to describe the next action, they usually pause the video and almost like put a color overlay to see better”). The participant also recommended using close-up camera views to show specific skills and suggested using photos and videos in the session, especially for non-native English or Portuguese speakers (“Another thing that they do with videos that I've noticed is, like when they're putting the pads on or whether opening, it's like very close. I guess camera views where, like, for example for this one is a good idea”). Inclusivity was a common theme as well, with all participants emphasizing the importance of providing voice-recorded questions for individuals who may have difficulty reading and suggesting alternative methods for testing ("...is to provide a training for people even people who do not can read"). The importance of providing training equipment and instructions that are simple and accessible was also highlighted. Additionally, the participants recommended using the same equipment during training that will be used in the field to make users more familiar with it (“Describing what equipment will be used, as simple as possible. Yeah. I totally agree”). The challenges of working in rural areas and areas of difficult access were also discussed, along with the importance of keeping personal information confidential (“People who are providing care to the women can share personal information with others, and ethical conflict here must be fixed”).

Content

Participant A provided positive feedback on the learning objectives but also asked for further explanation on AED operations (“How are they going to operate, right? That's this part of the video, right?”). Participant B provided feedback on the content and found no usability issues (“Just thinking, it's very good and don't need any comments”), while Participant C gave positive feedback on the methodical approach (“And to be honest, it's quite cool to see the consistencies across the board from International training to our training at all. All the evidence is the same so everyone's being taught the same thing”). Participants B and C also suggested adding more training equipment (Participant B: “Would you add more equipment to apply the training in real life?”, Participant C: “I think that more equipment is perfect"). Participant A found the content visually appealing and effective (“That's why using videos, figures, and other audio-visual aids, can make learning easier"), while Participants B and C believed that adding photos and graphics/drawings were essential for understanding the training (Participant B: “This would help people remember why you think it's more or less important”, Participant C: “Even more important for me is to have photos so I can continue”). Finally, Participant B emphasized ethical communication (“...this should emphasize the importance of being ethical with the information during the communication..."). Overall, the feedback was positive, with recommendations to add more real-life scenarios and training equipment, use a combination of photographs and graphics/drawings, and highlight ethical behavior when communicating with victims and sharing their information.

Design

Participant A did not provide any feedback related to the design in some of their responses. However, in one response, they suggested that the layout should be good and easy to visualize, which may indicate that they value a visually appealing and effective design (Participant B: “In training, graphic adjustment should be made, perhaps the organization or layout of the training”).

Participant B praised the design of the drone and equipment, saying it was very explanatory. They suggested adding space for the step-by-step process or just showing an overview of the training (“It makes sense logically to go through the step-by-step”). Additionally, they asked if recording the questions in the oral test instead of just text would be better (Participant B: “I think it's a very good idea. Would it make you more comfortable to take the course? I think so.”) They also inquired about the importance of training people to use acronyms during training for healthcare staff (Participant B: “I think it's important to train people to use acronyms during training, especially for healthcare staff”). They appreciated the way the training was presented and believed that the design of the equipment was very explanatory. 

Participant C suggested using different camera views to isolate specific skills and avoid causing anxiety in the viewer (“So being very thoughtful with the camera angles in the pauses to show each step of action because people want intuitively pick up on each”). Additionally, they suggested using a color overlay to highlight important information and pausing the video to describe critical points. They mentioned that the training equipment provided was perfect, and the AEDs were good for training purposes. However, they suggested that using a simulation AED or a similar training device can be more cost-effective and provide better training to the users (“…to be more familiar with the equipment. You're right, it doesn't have to be a real AED because it's expensive…”). They also suggested using graphics to cut out visual noise and direct the eye to what is important (“...one thing I've noticed with graphics, is it cuts out a lot of visual noise”). Finally, they suggested including a combination of photos and graphics to build recognition pathways while also providing clear and concise visual aids (“So having combination photos that are combined with graphics or combined with photo, I think would be good”). 

Overall, the participants' feedback suggests that the design of the maxSIMsaves (v2.0) training program is in general good and explanatory. However, there are some areas that can be improved, such as using clear audio, different camera views, color overlays, close-ups, and graphics to highlight important information. Participants also suggested adding space for the step-by-step process or just showing an overview of the training, as well as using a simulation AED or a similar training device to provide better training to the users. Finally, the participants suggested being very thoughtful with the camera angles and pausing to show each step of action in the video to help people intuitively pick up on each step and using visual aids to build recognition pathways while also providing clear and concise visual aids. Appendix B summarizes the analyzed data and their categories.

## Discussion

In this section, we will delve into the various contributions of the final maxSIMdrone version (Appendix C) using the EDVC approach. The development of this program has resulted in practical, methodological, and theoretical contributions that are worth exploring. In addition, limitations and future directions will be discussed in this session.

Practical contributions

The program aims to address critical gaps in training accessibility, communication methods, confidentiality, and inclusivity, benefiting individuals and communities in these R&R areas. These practical contributions are supported by studies that emphasize the need for accessible training programs and offline materials in resource-limited settings, the use of realistic simulations in remote areas, and the importance of confidentiality and privacy in healthcare training programs using online resources. By addressing the crucial aspects of training accessibility, communication, and inclusivity, this conceptual program aims to enhance emergency response capabilities in R&R areas, such as Amazon or the north of Canada. By providing a blueprint training program for training HCPs and LP to operate AEDs delivered by drones, the program seeks to contribute to improved survival rates in CA cases within these remote communities.

In Phase 1, the program's development focused on incorporating experts' feedback [22]. This phase emphasized offline content accessibility and using helicopters for realistic simulations and employing communication methods like radios and cell phones. By considering the limitations of internet access in R&R areas, the program ensures that training materials can be accessed offline [23]. Incorporating helicopters in simulations enhances the program's realism, enabling trainees to effectively operate AEDs in remote locations [24].

Phase 2, the hands-on practice phase, integrated experts' recommendations to improve the training program [20]. There were suggestions such as adding step-by-step videos and privacy instructions on communication through electronic platforms [25-26]. The involvement of a local resident as an information bridge addresses challenges of communication and ensures seamless coordination between health professionals and those providing assistance. Additionally, the program highlights the importance of confidentiality and includes training on maintaining patient data privacy [25].

During Phase 3, the feedback phase, the program was further refined based on participant input [16]. Video explanations highlighting correct and incorrect actions were incorporated to improve learning outcomes [26]. The iterative development process, combining participant feedback from earlier phases and TAOs, ensured that the final version of the program addressed usability, content, and design considerations [16].

Finally, studies have emphasized the need for accessible training programs and offline materials in resource-limited settings [23]. The use of helicopters or realistic simulators in simulations has been suggested as a way to provide realistic training experiences in remote areas [24]. Additionally, research highlights the importance of confidentiality and privacy in healthcare training programs using online resources [25]. Also, the maxSIMdrone program fills gaps in training accessibility, communication, and inclusivity. Its user-centered approach, incorporating participant feedback and scientific evidence, ensures that the program meets the needs of HCPs and LP operating AEDs delivered by drones in R&R areas. Additionally, by addressing these crucial aspects, the program aims to enhance emergency response capabilities, ultimately contributing to improved survival rates in CA cases within these remote communities.

Methodological contributions

Comparing and contrasting EDVC with other existing approaches, such as design-based research (DBR) and the approach proposed by Sivanathan et al., sheds light on the strengths and potential applications of EDVC [14].

EDVC differs from DBR in that it places a particular emphasis on incorporating qualitative participant feedback throughout the iterative development process [14]. In the case of maxSIMdrone, the MDT process in Phase 1 involved gathering valuable insights from participants and incorporating their suggestions and feelings into the program design [21-22]. This collaborative approach ensures that the training program aligns closely with the needs and preferences of the target audience.

Furthermore, the use of TAOs in Phase 3 of the EDVC approach adds an additional layer of feedback and expertise from specialists [22]. By involving specialists, such as the Brazilian doctor, nurse, and Canadian paramedic in the case of maxSIMdrone, the program benefits from their insights on usability, content, and design considerations [21]. This feedback-driven approach enhances the program's effectiveness and relevance. Furthermore, EDVC offers several advantages over alternative models and approaches. It combines the strengths of simulation, literature review, MDT, and TAOs, enabling a comprehensive and evidence-based program development process. By incorporating participant feedback and specialist input, EDVC ensures a user-centered approach that addresses the unique challenges of operating AEDs in R&R areas.

Finally, EDVC may be particularly suitable when developing training programs in resource-limited settings, where accessibility and relevance are paramount [27]. The emphasis on offline accessibility in Phase 1 and the inclusion of alternative testing methods for individuals with reading difficulties highlight EDVC's commitment to inclusivity. Additionally, the use of simulations, combined with participant and specialist feedback, strengthens the program's applicability and effectiveness in challenging environments [28].

Theoretical contributions

While the development of the maxSIMdrone training program using the EDVC approach primarily focuses on practical and methodological aspects, it also provides some theoretical contributions that can be linked to existing literature. Although there may not be extensive theoretical frameworks explicitly mentioned in the text, the program's design and implementation align with established educational and program design principles [11].

Phase 1 of the program development process, which emphasized "instruction", incorporated theories of adult learning and instructional design [9]. The participants' suggestions to include offline accessibility and real-life scenarios align with the principles of experiential learning [23-24]. By simulating authentic situations and providing hands-on experiences, the program caters to the needs of HCPs and LP operating AEDs in remote and rural areas [10].

In Phase 2, the hands-on practice phase, the incorporation of participants' recommendations reflects the principles of overarching theory [9]. The emphasis on simplicity and the inclusion of step-by-step videos and pictures enhance understanding and knowledge retention [9]. By involving a local resident as an information bridge, the program promotes social learning and collaborative problem-solving [29].

Phase 3, the feedback phase involving TAOs, aligns with the principles of user-centered design and usability testing [16]. The participants' feedback on usability, content, and design considerations demonstrates the importance of user feedback in the iterative development process [22]. The incorporation of video explanations to highlight correct and incorrect actions reflects the principles of cognitive load theory, as it provides learners with visual cues and demonstrations [26,30].

The program's focus on inclusivity, accessibility, and privacy considerations aligns with principles of ethics in educational and healthcare settings [25]. By addressing the challenges faced in remote and rural areas, the program also contributes to the existing literature on healthcare access and training in underserved regions [27].

In summary, while the study does not provide explicit references to specific theoretical frameworks, the principles and concepts discussed in the program's development process can be linked to theories of adult learning, experiential learning, user-centered design, cognitive load theory, equity, and ethics [9,16,23,24,30]. These connections highlight the program's alignment with existing literature and educational and program design principles [12].

Limitations

The maxSIMdrone training program, despite its contributions, has certain limitations. Firstly, its focus on R&R areas may limit its applicability in urban or densely populated regions, where the challenges and dynamics of emergency response may differ. Secondly, the program's implementation requires significant resources, including access to drones, AEDs, and trained personnel, which may pose challenges in resource-limited settings. Additionally, cultural adaptation could be a potential limitation, as the program's design and content may need to be tailored to specific cultural contexts to ensure optimal effectiveness and acceptance. Furthermore, the program's reliance on participant feedback and specialist input may introduce bias or overlook certain perspectives, potentially limiting its inclusivity and comprehensiveness. Lastly, the program's effectiveness and long-term impact on survival rates in CA cases in remote communities need to be evaluated through rigorous monitoring and evaluation frameworks. Addressing these limitations would strengthen the program's scalability, adaptability, and overall effectiveness in diverse settings, ensuring a broader impact in improving emergency response and healthcare outcomes.

Future directions

As a prototype, the maxSIMdrone training program holds promising potential for future development and implementation. Building upon its practical and methodological contributions, several directions can be explored to further enhance the program's effectiveness and address evolving needs.

In terms of practical contributions, future iterations could incorporate advanced technologies, such as artificial intelligence and virtual reality, to provide more immersive and interactive training experiences. Integration with emerging communication platforms and devices, such as drones and wearable technology, can further enhance communication methods and expand the program's reach. Methodologically, continued collaboration with participants and specialists will ensure ongoing refinement and adaptation to changing contexts. Expanding the scope of research to encompass a wider range of resource-limited settings and diverse populations will contribute to the program's inclusivity and relevance.

From a theoretical standpoint, maxSIMdrone may involve exploring additional educational and program design principles, such as transformative learning and sociocultural theories, to deepen the program's impact. Emphasizing the ethical considerations of healthcare training and addressing equity issues will remain crucial focal points.

Overall, the maxSIMdrone program involves leveraging emerging technologies, engaging in ongoing collaboration, and exploring additional theoretical frameworks. By continuously evolving and adapting, the program can effectively contribute to improving emergency response capabilities and saving lives in R&R areas.

## Conclusions

In conclusion, this research study presents an overview of the EDVC approach design used to develop and validate the maxSIMdrone program for HCPs and LP to provide out-of-hospital CA care using AEDs delivered by drones in R&R locations. The article highlights the importance of using the MDT process and TAO methodology to gather feedback from participants and create a comprehensive training program. The participants recommended changes to the online training model, including making the program offline accessible, using a helicopter as a means of transport, and adding oral tests to the written tests. In the hands-on practice phase, participants suggested adding step-by-step videos and information on how to call for help through various means. The feedback phase included video explanations to help participants understand how to improve their performance. 

Overall, the final maxSIMdrone program incorporates the suggestions provided by participants and offers a comprehensive training program to provide out-of-hospital CA care using AEDs delivered by drones. The program is an essential step in improving knowledge and techniques on communication and instruction to HCPs and LP in the proper use of AEDs, especially for people in R&R locations with limited resources.

## Appendices

Appendix A 

**Table 2:** Participants' Opinions in Usability, Content, and Design for TAO Methodology. AED: automatic external defibrillator EMS: emergency medical service LMS: learning management system R&R: rural and remote

Task	Participant	Usability Feedback	Content	Design
#1	Participant A	The participant does not provide any feedback related to usability.	The participant provides feedback on the learning objectives presented in Phase 1, highlighting its level of detail and quality, and how it serves people well. They give an example of how a specific topic is explained in easy words, making it easier for day-to-day use. They also ask for further explanation about how it should be and how AED will operate.	The participant does not provide any feedback related to design.
	Participant B	Based on the participant's responses, it seems that they did not have any usability issues with the learning objectives presented in Phase 1. However, they did comment on the content and mentioned that they found it to be "really good."	The response can be divided into 1 - no usability issues, 2 - positive feedback on content.	The participant does not provide any feedback related to design.
	Participant C	The participant does not provide any feedback related to usability.	The participant provided positive feedback on the content of Phase 1, noting that it was methodical and did a good job of starting at a high level before diving into the details of how to use the technology. They appreciated the focus on accessing the technology and considering the resources of the audience. The participant also noted that they liked the tie-in to survival rates and consistency with other programs.	The participant does not provide any feedback related to design.
#2	Participant A	The participant seems to suggest that the interface must be easy to use.	The participant seems to suggest that the interface must be easy to use.	The participant seems to suggest that the general layout should be good and easy to visualize.
	Participant B	The participant suggests adding more training, specifically using a mannequin for practical training. The participant also suggests adding an oral test for people who may not be able to read. The participant suggests using acronyms during training for healthcare staff.	The participant expresses that the videos are helpful for those who do not know how to work with defibrillators and they are presented well. The participant asks if the videos should have space for a step-by-step process or just show an overview of the training, and suggests that more training be added for real-life events.	The participant praises the design of the drone and equipment, saying it is very explanatory. The participant asks if recording the questions in the oral test instead of just text would be better.
	Participant C	The participant suggested that the video should focus on critical points where the next action needs to be described. They also recommended pausing the video and using a color overlay to highlight important information. The participant also mentioned the importance of having clear audio and avoiding background noise. Additionally, they suggested having close-up camera views to show specific skills, such as how to unclip the box, open it, turn on the device, and put the pads on properly.	The participant suggested that the video should cover all fail points and focus on specific skills to avoid responder failure. For instance, they recommended showing how to open the box, how to pick up the drone without cutting oneself, and how to place the pads correctly. They also suggested that the video should be useful for a layperson who might not be familiar with medical procedures.	The participant mentioned that the current video did not have clear audio due to the background noise of the drone. They also suggested using different camera views to isolate specific skills and avoid causing anxiety in the viewer. Additionally, they suggested using a color overlay to highlight important information and pausing the video to describe critical points.
#3	Participant A	The participant seems to appreciate the simplicity of the training equipment and text, which is important for people with limited education and understanding. The participant suggests that drawings and figures may be easier to interpret than text.	The participant mentions that the drawing is wonderful, which suggests that the content is visually appealing and effective. However, the participant does not provide any specific feedback on the content of the training equipment or text.	The participant does not provide any feedback specifically related to the design of the training equipment but mentions that the drawing is wonderful, which may suggest that the design is visually appealing and effective.
	Participant B	The participant suggests that some people may not have adequate training and more equipment should be added to apply the training in real-life scenarios. The participant suggests adding a mannequin to the training equipment. The participant suggests including oral tests for indigenous health and people who don't speak Portuguese. The participant suggests using acronyms during training for healthcare staff.	The participant thinks it's important for those who still don't know how to work with defibrillators to have training equipment. The participant suggests adding more training equipment, including a phone, a tablet, and the protocol itself.	The participant thinks the training equipment's presentation is good. The participant suggests adding space for the step-by-step process or just showing an overview of the training.
	Participant C	The iPad provided is user-friendly and provides access to instructional videos. The reusable button shirts should be used during the training to simulate real-life scenarios effectively. It is important to use the same equipment during training that will be used in the field to make users more familiar with it.	The mannequins used during training are effective, but reusable button shirts should be used instead of naked mannequins or button shirts that can easily be ripped off. The training equipment provided should be capable of simulating real-life scenarios effectively to train EMS to operate drone-delivered AEDs at R&R sites.	The training equipment provided is perfect, and the provided AED is good for training purposes. However, using a simulation AED or a similar training device can be more cost-effective and provide better training to the users.
#4	Participant A	Based on the participant's response, it does not provide any feedback related to the usability of the voice-recorded questions. The participant instead provides an opinion on the importance of making training accessible and suggests using audio-visual aids to make learning easier.	Based on the participant's response, there is no indication of any issues related to the content of the training. The participant seems satisfied with the course content and does not suggest any changes or improvements. Therefore, there is no feedback related to the content category.	The participant did not provide any feedback related to design.
	Participant B	The participant emphasized the importance of providing voice-recorded questions for individuals who may have difficulty reading. They suggested adding more training, specifically with a mannequin, to better prepare participants for real-life situations. They recommended including oral tests for indigenous health and using acronyms during training for healthcare staff.	The participant asked about the form of the video and whether it should show a step-by-step process or just an overview of the training. They mentioned that the subjects addressed in the training were dynamic and that the design of the drone equipment was explanatory.	The participant asked about the format of the test after watching the learning objectives and material, specifically whether it should be recorded as just text or also include voice recordings. They inquired about the importance of training people to use acronyms during training for healthcare staff.
	Participant C	The participant raises the issue of inclusivity in the training for people who cannot read or write in the primary language of the training. They suggest providing a voice-over option for the graphics-heavy videos to make them more accessible and inclusive. They also suggest including a voice-recorded question feature at the end of the training, which can be answered verbally instead of writing.	The participant suggests creating a second set of videos with voice-overs in different languages to make the training more accessible and inclusive for people from different cultures. They also suggest breaking down the graphics into phases as someone is talking, so people can get a visual aspect as well as auditory to improve their understanding of the training.	The participant suggests being very thoughtful with the camera angles and pauses to show each step of action in the video to help people intuitively pick up on each step. They also suggest using close-ups to show each step of action and having very specific intuitive jumps throughout the video to be helpful.
#5	Participant A	The participant suggests that it's important to present instructions as simply as possible for those who may struggle with complex instructions. They also mention that drawings and figures can be easier to interpret than text, which could improve usability.	The participant appreciates the use of drawings and figures in the training session, finding them valuable and easier to interpret than text. They also mention that the equipment depicted in the drawing is all there, indicating that the content is comprehensive.	The participant does not provide specific feedback related to design in this response.
	Participant B	The participant suggests adding more training equipment, such as a mannequin, to improve the training's effectiveness. They recommend including oral tests in addition to written tests to cater to those who cannot read or speak Portuguese. The participant suggests using acronyms during training to help healthcare staff identify patients more easily.	The participant believes that including photographs in the session is crucial for those who don't know how to work with defibrillators, as it helps them understand how it happens. They recommend adding more content to the training for those who don't have adequate training.	The participant appreciates the way the training is presented and believes that the design from the drone of the equipment is very explanatory. They suggest providing space for the step-by-step process or just showing an overview of the training.
	Participant C	The participant suggests that including photos and videos in the session is crucial, especially for non-native English speakers. They mention that having photos can help them keep up with what is going on in the session.	The participant talks about the importance of having a combination of photos and graphics/drawings to aid in recognition pathway building. They also suggest including photos that are combined side by side or in natural settings to better illustrate the content being presented.	The participant mentions that using graphics can cut out visual noise and direct the eye to what is important. They suggest including a combination of photos and graphics to build recognition pathways while also providing clear and concise visual aids.
#6	Participant A	The participant mentioned the possibility of communication failure and the need for a third person to act as a communicator source. The participant also highlighted the importance of considering the challenges of working in rural areas and areas of difficult access.	The participant expressed interest in the scenario being realistic and highlighting the challenges that may occur in communication. The participant also emphasized the importance of being ethical with the victims' information during communication.	There was no feedback from the participant that specifically relates to design.
	Participant B	The participant suggests adding more training, specifically using a mannequin for practicing. They also suggest including oral tests for indigenous health and using recordings to help those who cannot read. The participant recommends using acronyms during training for healthcare staff to identify patients.	The participant mentions the importance of emphasizing ethical behavior while communicating and handling the victims' information. They also suggest highlighting the protocol for using the defibrillator and drone in the training.	The participant appreciates the design of the training, particularly the explanatory design of the drone equipment. They ask if the video format is sufficient for the step-by-step process or if it should show an overview of the training.
	Participant C	The participant mentions that sometimes the victim is far away from the healthcare professional, and there can be a breach in communication via radio. There is an ethical conflict because important personal information should not be shared with everyone. The participant suggests that during the training, an emphasis should be placed on the importance of keeping the information tight and not sharing personal information, including a recommendation for what information needs to be relayed.	The participant asks if there is a recommendation or a protocol for what information needs to be relayed. They suggest using a graphic, such as an infant picture, to indicate age or size of the patient. They mention that they do not care about the name during dispatching, but they care about other details like age, sex, and size of the patient.	The participant suggests using a laminated flashcard during training to emphasize the importance of being ethical with the victims' information during communication and highlight that other people not involved in the service can hear all the communication.
#7	Participant A	Based on the participant's responses, it seems that they did not directly address the question about the option of recording videos and sending them to receive feedback. Instead, they expressed interest in the scenario being realistic and reflecting the challenges of working in rural areas with limited access to technology and literacy.	Therefore, the content category might be the most appropriate for this feedback. The participant's response highlights the importance of considering the context in which the training will be delivered and ensuring that the scenario is relevant and relatable to the target audience.	The participant does not provide specific feedback related to design in this response.
	Participant B	The option of recording videos and sending them for feedback is helpful for those who cannot read or write. Some people may not have adequate training, and it would be good to add more. The participant suggests adding a mannequin for training purposes. Oral tests should be included for indigenous health, and it would be helpful to have them in a recording format for those who cannot read.	The participant thinks it's important to have a step-by-step process in the video, or at least an overview of the training. The participant suggests adding more equipment for real-life training scenarios. The participant suggests using acronyms to identify patients during training for healthcare staff.	The participant thinks the design of the drone and equipment is explanatory and dynamic.
	Participant C	The participant suggests using visual aids to communicate as it may be challenging to communicate using written language. The participant also suggests trialing the training with a group that does not speak the language to identify comprehension fail points and improve the training's layout and organization.	The participant does not provide any specific feedback related to the content of the training.	The participant suggests improving the training's design by using visual aids and improving the layout and organization based on the comprehension fail points identified during the trial.
#8	Participant A	The participant does not mention any specific usability issues with the LMS or online simulation-based training model. However, they suggest using videos, figures, and other audio-visual aids to make learning easier, especially in places where accessing buildings is difficult.	The participant does not provide any feedback on the content of the course.	The participant is satisfied with the course's design and does not suggest any changes.
	Participant B	The course is easy to navigate. The instructions and materials are clear and concise. Oral tests should be included for indigenous health and patients who don't speak Portuguese. Acronyms should be used during training, especially for healthcare staff.	The online simulation-based training model is helpful for those who don't know how to work with defibrillators. More training should be added, such as using a mannequin. A test should be included after watching the video to assess knowledge.	The video format is good, but the participant asks if it should include space for step-by-step processes or just show an overview of the training. The design of the drone equipment is explanatory. A mannequin should be added for training purposes.
	Participant C	The course is easy to navigate. Graphics help in navigating the course. The instructions are clear and concise.	The material is concise and focused on learning objectives. There is no unnecessary information.	The overall structure and design of the LMS and online simulation-based training model are good The design is well thought out and considers the target audience and their resources, including language and communication resources.

Appendix B 

**Table 3:** The maxSIMdrone (v1.0) and (v2.0) With Notes From Researchers and Experts' Opinions. AED: automatic external defibrillator BLSD: basic life support and defibrillation CA: cardiac arrest CPR: cardiopulmonary resuscitation LMS: learning management system PHOENIX: drone VF/VT: ventricular fibrillation/ventricular tachycardia 911: emergency service Y: yes N: no

maxSIMdrone (v1.0)			-	maxSIMdrone (v2.0)		
Phase 1: Proficiency-based development of actions representations			Notes/Comments	Phase 1: Proficiency-based development of actions representations		
1 - Phase 1: Proficiency-based development of action representations (Instruction). Learning objectives.			Notes: 1 - Must be offline as well. Alpha comments: “We have difficulty accessing the internet, so I believe the information must be offline as well". Beta comments: I agree with "Alpha" the information needs to be offline if we don't have internet access.	1 - Phase 1: Proficiency-based development of action representations (Instruction). Learning objectives: There are five learning objectives that participants are expected to learn in this course. In addition, the material must also be accessed offline.		
1 - Guidance about safety: The participants must ensure that the site is safe for the rescuer and the victim so that he or she does not become the next victim. If the location is safe, then care of the patient can safely continue. 2 - Guide assessing the victim's responsiveness: The participants should guide how the victim's responsiveness should be assessed by calling out and shaking their shoulders. If the victim responds, participants should guide the rescuer to talk to the victim and ask if he or she needs help. If the victim does not respond, immediately send the drone and helicopter. 3 - Send help (drone and ambulance): In an extra-hospital environment, participants must immediately send the PHOENIX with an AED and a helicopter with a team of paramedics. At the same time, participants must advise on resuscitation maneuvers. It is important to designate people to be responsible for performing these functions. 4 - Guide to checking breathing and pulse: If the victim does not breathe or gasps and the pulse is absent, participants should promptly initiate cardiopulmonary resuscitation. 5 - Guide start cycles of 30 compressions and two ventilations: Participants should guide the start cycle of 30 compressions and two ventilations, considering that there is a barrier device (for example, a pocket mask to apply the ventilation).			Notes: 1 - The online training model must be able to be accessed offline as well. 2 - Health professionals asked that the means of transport that should be sent in the training be a helicopter to approximate what happens in the real world. 3 - The means of communication between the provider and the health professional must be radio and cell phone with internet. Alpha comments: “This session needs to be offline too”, “We use radios as well but sometimes WhatsApp app when we have internet access.” Gamma comments: ‘I think It's good to exchange an ambulance for a helicopter because we use helicopters here.” Beta comments: “We use radio and cell phones, add radios here too.”	1 - Guidance about safety: Via radio, a call cellphone, or WhatsApp app, the participants must ensure that the site is safe for the rescuer and the victim so that he or she does not become the next victim. If the location is safe, then care of the patient can safely continue. 2 - Guide assessing the victim's responsiveness: The participants should guide how the victim's responsiveness should be assessed by calling out and shaking their shoulders. If the victim responds, participants should guide the rescuer to talk to the victim and ask if he or she needs help. If the victim does not respond, immediately send the drone and a helicopter. 3 - Send help (drone and helicopter): In an extra-hospital environment, participants must immediately send the PHOENIX with an AED and a helicopter with a team of paramedics. At the same time, participants must advise on resuscitation maneuvers. It is important to designate people to be responsible for performing these functions. 4 - Guide to checking breathing and pulse: If the victim does not breathe or gasps and the pulse is absent, participants should promptly initiate cardiopulmonary resuscitation. 5 - Guide start cycles of 30 compressions and two ventilations: Participants should guide the start cycle of 30 compressions and two ventilations, considering that there is a barrier device (for example, a pocket mask to apply the ventilation).		
2 - Phase 1: Proficiency-based development of action representations (Instruction). Video demonstration of PHOENIX delivering AED and provider receiving instruction over the phone. VIDEO			Notes: 1 - Instructional videos should be recorded in a remote environment to show what participants can expect from the training. The videos must contain all the walkthroughs of the BLSD stages in the lifesaving process and how to use an AED. Alpha comments: ”I think the video should be showing how to do CPR and use the AED too and all equipment as well.” Gamma comments: “I think the video should be recorded inside the forest so people already know how it would be. It would be nice to see a video recorded in a remote location.”	2 - Phase 1: Proficiency-based development of action representations (Instruction). Video demonstration of PHOENIX delivering AED and provider receiving instruction over the phone. The videos that must be in the instruction phase must contain an instructor explaining in detail each step-by-step of the training, as well as how to operate an AED and the entire BLSD stages in the lifesaving process. VIDEO		
3 - Phase 1: Proficiency-based development of action representations (Instruction). The stages in the lifesaving process for AEDs delivered by drones. The stages in the lifesaving process. The stages in the lifesaving process are a series of steps that can be taken to improve the chances of survival for someone experiencing sudden cardiac arrest. The steps in the stages in the lifesaving process are: 1 - Recognition and activation of the emergency response system: The first step in the lifesaving process is to recognize that someone is experiencing sudden cardiac arrest and to activate the emergency response system by calling 911. This should be done as soon as possible, as time is of the essence in these situations. 2 - Call 911: Before starting CPR, the witness should call 911, ask for help, and when instructed he or she should start the intervention. 3 - The healthcare professional sends help and provides guidance: The healthcare professional who receives the call from the witness must immediately give instructions on how to identify if the victim is breathing and if he or she has an arterial pulse. If the healthcare professional believes that the case is a cardiac arrest, he or she should immediately request the dispatch of a drone with an AED and a team of paramedics by helicopter when available. 4 - Start high-quality CPR: Initiate high-quality CPR as instructed by the healthcare professional until the AED delivered by drone or helicopter arrives. This involves chest compressions and rescue breaths to keep oxygen-rich blood circulating to the brain and other vital organs. Anyone who is trained in CPR should be able to provide this life-saving intervention. 5 - Rapid Defibrillation: The fifth step in the lifesaving process is to use a defibrillator, or AED, to deliver a shock to the heart. This can help to restore a normal heart rhythm and improve the chances of survival for someone experiencing sudden cardiac arrest. AEDs will be delivered by a drone. 6 - Advanced Life Support: The sixth step in the lifesaving process is to provide advanced life support, such as medications and other interventions, to help restore normal heart function and stabilize the person’s condition. This is typically done by paramedics or other medical professionals who are trained in advanced life support techniques. 7 - Post Arrest Care: The final step in the lifesaving process is to provide post-arrest care, such as monitoring and support for the person’s heart and other vital functions, to help them recover from the cardiac arrest. This may involve hospitalization and specialized treatment, such as coronary angiography or coronary artery bypass surgery. By following the step in the lifesaving process, it is possible to improve the chances of survival for someone experiencing sudden cardiac arrest. It is important to act quickly and provide the appropriate interventions to help save a life.			1 - No changes suggested.	3 - Phase 1: Proficiency-based development of action representations (Instruction). The stages in the lifesaving process for AEDs delivered by drones. The stages in the lifesaving process. The stages in the lifesaving process is a series of steps that can be taken to improve the chances of survival for someone experiencing sudden cardiac arrest. The steps in the stages in the lifesaving process are: 1 - Recognition and activation of the emergency response system: The first step in the lifesaving process is to recognize that someone is experiencing sudden cardiac arrest and to activate the emergency response system by calling 911. This should be done as soon as possible, as time is of the essence in these situations. 2 - Call 911: Before starting CPR, the witness should call 911, ask for help, and when instructed he or she should start the intervention. 3 - The healthcare professional sends help and provides guidance: The healthcare professional who receives the call from the witness must immediately give instructions on how to identify if the victim is breathing and if he or she has an arterial pulse. If the healthcare professional believes that the case is a cardiac arrest, he or she should immediately request the dispatch of a drone with an AED and a team of paramedics by helicopter when available. 4 - Start high-quality CPR: Initiate high-quality CPR as instructed by the healthcare professional until the AED delivered by drone or helicopter arrives. This involves chest compressions and rescue breaths to keep oxygen-rich blood circulating to the brain and other vital organs. Anyone who is trained in CPR should be able to provide this life-saving intervention. 5 - Rapid Defibrillation: The fifth step in the lifesaving process is to use a defibrillator, or AED, to deliver a shock to the heart. This can help to restore a normal heart rhythm and improve the chances of survival for someone experiencing sudden cardiac arrest. AEDs will be delivered by a drone. 6 - Advanced Life Support: The sixth step in the lifesaving process is to provide advanced life support, such as medications and other interventions, to help restore normal heart function and stabilize the person’s condition. This is typically done by paramedics or other medical professionals who are trained in advanced life support techniques. 7 - Post Arrest Care: The final step in the lifesaving process is to provide post-arrest care, such as monitoring and support for the person’s heart and other vital functions, to help them recover from the cardiac arrest. This may involve hospitalization and specialized treatment, such as coronary angiography or coronary artery bypass surgery. By following the step in the lifesaving process, it is possible to improve the chances of survival for someone experiencing sudden cardiac arrest. It is important to act quickly and provide the appropriate interventions to help save a life.		
4 - Phase 1: Proficiency-based development of action representations (Instruction). Recommended Equipment: 1 - PHOENIX: This equipment will be used to simulate the drone controller in real time. 2 - One AED: This equipment will be used to simulate cardiac arrest treatment in which the patient would be presenting ventricular tachycardia or ventricular fibrillation. 3 - A mannequin: This simulator will be used to simulate a cardiac arrest victim. 4 - Two radios: This equipment will be used for remote communication between the provider and the healthcare professional.			Notes: 1 - It must have photos of all kinds of equipment as well as radio and cell phones and pictures. Gamma comments: “Add radios photo here too." Delta comments: “I think not just pictures of radios, but should have pictures of all equipment.”	4 - Phase 1: Proficiency-based development of action representations (Instruction). Recommended Equipment: 1 - PHOENIX: This equipment will be used to simulate the drone controller in real time. 2 - One AED: This equipment will be used to simulate cardiac arrest treatment in which the patient would be presenting ventricular tachycardia or ventricular fibrillation. 3 - A mannequin: This simulator will be used to simulate a cardiac arrest victim. 4 - Two radios or cellphones: This equipment will be used for remote communication between the provider and the healthcare professional. 5 - Rescue team simulating that it arrived in a helicopter. Photo: Cellphones, tablets, AED, radio, drone, and stages in the lifesaving process.		
5 - Phase 1: Proficiency-based development of action representations (Assessments). To move on to the next phase, the learners must mark the action option as "very important" for all options. 1 - Take the phone call and identify yourself as a healthcare professional. Not important ( ) Important ( ) Very important ( ) 2 - Guidance about safety. Not important ( ) Important ( ) Very important ( ) 3 - Guidance on assessing the victim's responsiveness and breathing. Not important ( ) Important ( ) Very important ( ) 4 - Sending help! Not important ( ) Important ( ) Very important ( ) 5 - Guide to checking breathing and pulse: Guidance on checking the pulse and starting cardiopulmonary resuscitation if the pulse is absent or if he or she is in doubt. Not important ( ) Important ( ) Very important ( ) 6 - Guide to start cycles of 30 compressions and two ventilations: Guide the completion of three cycles of continuous compressions (200) with passive oxygen ventilation, in cases of cardiac arrest witnessed with VF/VT rhythm. I: Guide about the positioning of the hand: Rock the heel of the hand off the chest, keeping fingertips on the chest wall to maintain hand position. II: Chest compressions must have a minimum depth of 5 cm, without exceeding 6 cm. III: The delay of compressions between shock delivery must be as short as possible. IV: Resume chest compressions immediately after defibrillation for adults in cardiac arrest. Not important ( ) Important ( ) Very important ( ) 7 - Performing 30 compressions and two ventilations, for adults in cardiac arrest. I: It is advisable to perform compressions at a frequency of 100 to 120 compressions/minute. II: Chest compressions must have a minimum depth of 5 cm, without exceeding 6 cm. III: The delay of compressions between shock delivery must be as short as possible. IV: Resume chest compressions immediately after defibrillation for adults in cardiorespiratory arrest. Not important ( ) Important ( ) Very important ( ) 8 - Guide to maintaining the cardiopulmonary resuscitation maneuvers and the AEDs analysis until the paramedic team arrives at the scene and takes over or until the victim shows signs of life. Not important ( ) Important ( ) Very important ( ) 9 - Guide to maintaining the cardiopulmonary resuscitation maneuvers and the AEDs analysis until the paramedic team arrives at the scene and takes over or until the victim shows signs of life. Not important ( ) Important ( ) Very important ( ) 10 - The participant must now instruct the woman that her father will be taken to a hospital by the paramedic team. Not important ( ) Important ( ) Very important ( )			Notes: 1 - In addition to a written test, this test should be an oral test (voice record for the test questions and for the participant to upload) because some lay people cannot read and write very well. Alpha comments: “I believe it should have audio, some people can't read." Beta comments: “Yes, definitely should have questions in audio format.”	5 - Phase 1: Proficiency-based development of action representations (Assessments). To move on to the next phase, the learners must mark the action option as "very important" for all options. 1 - Take the phone call and identify yourself as a healthcare professional. Not important ( ) Important ( ) Very important ( ) 2 - Guidance about safety. Not important ( ) Important ( ) Very important ( ) 3 - Guidance on assessing the victim's responsiveness and breathing. Not important ( ) Important ( ) Very important ( ) 4 - Sending help! Not important ( ) Important ( ) Very important ( ) 5 - Guide to checking breathing and pulse: Guidance on checking the pulse and starting cardiopulmonary resuscitation if the pulse is absent or if he or she is in doubt. Not important ( ) Important ( ) Very important ( ) 6 - Guide to start cycles of 30 compressions and two ventilations: Guide the completion of three cycles of continuous compressions (200) with passive oxygen ventilation, in cases of cardiac arrest witnessed with VF/VT rhythm. I: Guide about the positioning of the hand: Rock the heel of the hand off the chest, keeping fingertips on the chest wall to maintain hand position. II: Chest compressions must have a minimum depth of 5 cm, without exceeding 6 cm. III: The delay of compressions between shock delivery must be as short as possible. IV: Resume chest compressions immediately after defibrillation for adults in cardiac arrest. Not important ( ) Important ( ) Very important ( ) 7 - Performing 30 compressions and two ventilations, for adults in cardiac arrest. I: It is advisable to perform compressions at a frequency of 100 to 120 compressions/minute. II: Chest compressions must have a minimum depth of 5 cm, without exceeding 6 cm. III: The delay of compressions between shock delivery must be as short as possible. IV: Resume chest compressions immediately after defibrillation for adults in cardiorespiratory arrest. Not important ( ) Important ( ) Very important ( ) 8 - Guide to maintaining the cardiopulmonary resuscitation maneuvers and the AEDs analysis until the paramedic team arrives at the scene and takes over or until the victim shows signs of life. Not important ( ) Important ( ) Very important ( ) 9 - Guide to maintaining the cardiopulmonary resuscitation maneuvers and the AEDs analysis until the paramedic team arrives at the scene and takes over or until the victim shows signs of life. Not important ( ) Important ( ) Very important ( ) 10 - The participant must now instruct the woman that her father will be taken to a hospital by the paramedic team. Not important ( ) Important ( ) Very important ( )		
Phase 2: Home-based and self-directed practice				Phase 2: Home-based and self-directed practice		
6 - Phase 2: Home-based and self-directed practice (Hands-on practice). Once proficient action representations have been formed, the learners move to Phase 2 - Here, they would practice BLSD using the PHOENIX and AEDs specifically designed for this purpose and the stages in the lifesaving process.			Notes: 1 - Add walkie-talkie pictures. 2 - Add a photo from a tablet or cell phone with step-by-step videos on how to carry out the training and which will also serve to film the training to upload for feedback in phase. Alpha comments: “Explain here how people should call for help using radio, phone, or WhatsApp." Gamma comments: “You could keep pictures of all equipment here as well.” Delpa comments: “I think that at the time of training, a tablet or cell phone with instructional videos should be available for the participant to watch when he or she feels unable to perform the training correctly.”	6 - Phase 2: Home-based and self-directed practice (hands-on practice). Once proficient action representations have been formed, the learners move to Phase 2 - Here, they would practice BLSD using the equipment specifically designed for this purpose and the stages in the lifesaving process (radios, cellphones, iPads). Photo: Cellphones, tablets, AED, radio, drone, and stages in the lifesaving process.		
7 - Phase 2: Home-based and self-directed practice (Hands-on practice). Inputs - Equipment: This simulation can be performed in a controlled environment using a mannequin or an actor. A camera on a tripod, speakers, and microphones can play the role of a drone at the service point in addition to an AED. This simulation is intended for health professionals who operate within an urgent or emergency network such as 911 where there is a trained healthcare professional who can assist and guide patients and victims by telephone or radio. This simulation will be performed in both urban and rural or remote places where a drone reaches the victim first but a road ambulance is delayed. Information: Ideally, the simulation is aimed at healthcare professionals who work in emergency care such as 911 in urban or rural areas, or who seek additional training in the treatment of patients with cardiac arrest. As the simulation takes place in a prehospital environment, there is no access to imaging or laboratory investigations. Facilitators: Two health professionals, both comfortable in providing care for patients with CA, should act as facilitators. These professionals must have experience with the use of AEDs. One should be designated as the primary facilitator, guiding participants and helping with the overall organization and execution of the case, while the second facilitator will be present to assess individual performance. Facilitators are responsible for providing participants with the appropriate information as requested. Facilitators should examine the scenario in advance to identify possible limitations or technical problems related to the proper functioning of the camera, speakers, microphone, and AEDs. Context: Participants initiate the case in a 911 urgent and emergency care service, which contains a physician and a licensed drone pilot (the latter can be interpreted by the facilitator). The phone rings and a 25-year-old woman is desperate and crying a lot on the phone. She is camping with her 55-year-old father and reports that three minutes ago he screamed with a pain in his chest and simply fell to the ground. She believes that he is not breathing. Prebriefing: Before starting the simulation, participants must be submitted to the fiction contract, which recognizes that everything taking place during the simulation must be treated as if they were "real" so that the objectives of the simulation can be achieved. During this period, the facilitators present the simulation scenario and all the necessary precautions, before presenting the AED, the simulation scenario with the camera, speakers, and microphones, and describe the function of each piece of equipment. If there is a limited supply of participants, the paramedic’s team can only be informed by the facilitators, as the main objective of this simulation is the fundamental aspects of BLSD in adults, which include immediate recognition of CA, contact with the emergency system, initiation of high-quality cardiopulmonary resuscitation, and use of AED as soon as possible.			Notes: 1 - Add information that people will call for help or WhatsApp message or walkie-talkie. 2 - The scenario must be as simple as possible and, in addition to a text, it must have an audio description so that there is no difficulty in understanding. 3 - It should emphasize the importance of preserving patient data during training. This should form part of the training so that participants understand the importance of this. 4 - It should include information that in some situations the radios are very far away and a third person, usually a local resident who is between the two, can serve as an information bridge between the health professional and who is providing assistance to the victim with cardiac arrest. Alpha comments: “Put that people sometimes use the WhatsApp application to ask for help when they have access to the internet.”, “The scenario has to be the simplest and easiest. Some people have difficulty following complex scenarios.”, "Put the information here that sometimes the radios of the health professional and the provider are far away and in some cases, a resident who has a radio and is between the two can serve as a bridge of information between them.” Beta comments: "I think you should also talk about preserving victim information here. Sometimes they say the name of the victim and what is happening to them and everyone who has a radio can identify these victims.", "Patient information must be confidential and participants need to train this as well." Delta comments: “The text here could also have audio, it is easier to understand.”	7 - Phase 2: Home-based and self-directed practice (Hands-on practice). Inputs - Equipment: This simulation can be performed in a controlled environment using a mannequin or an actor, one iPad to follow video instructions, radios or cellphones for communication, a PHOENIX, and AED. This simulation is intended for health professionals who operate within an urgent or emergency network such as 911 where there is a trained healthcare professional who can assist and guide patients and victims by telephone or radio. This simulation will be performed in a rural or remote place where a drone reaches the victim first but a helicopter is delayed. Information: Ideally, the simulation is aimed at healthcare professionals who work in emergency care such as 911 in urban or rural areas, or who seek additional training in the treatment of patients with cardiac arrest. As the simulation takes place in a prehospital environment, there is no access to imaging or laboratory investigations. Facilitators. Two health professionals, both comfortable in providing care for patients with CA, should act as facilitators. These professionals must have experience with the use of AEDs. One should be designated as the primary facilitator, guiding participants and helping with the overall organization and execution of the case, while the second facilitator will be present to assess individual performance. Facilitators are responsible for providing participants with the appropriate information as requested. Facilitators should examine the scenario in advance to identify possible limitations or technical problems related to the proper functioning of the radios or cellphones, iPad, PHOENIX, and AEDs. Before training begins, facilitators should explain the importance of not identifying the victim while training is taking place. This must be reinforced at all times during the program. Context: Participants initiate the case in a 911 urgent and emergency care service, which contains a healthcare professional and a licensed drone pilot (the latter can be interpreted by the facilitator). The phone rings by call or WhatsApp or a call from a radio is initiated and a 25-year-old woman is desperate and crying a lot on the call. She is camping with her 55-year-old father and reports that three minutes ago he screamed with a pain in his chest and simply fell to the ground. She believes that he is not breathing. If it is by radio, sometimes the communication may fail and a third person will have the role of being a communication bridge between the woman and the health professional because this person gets a good signal from both. Prebriefing: Before starting the simulation, participants must be submitted to the fiction contract, which recognizes that everything taking place during the simulation must be treated as if they were "real" so that the objectives of the simulation can be achieved. During this period, the facilitators present the simulation scenario and all the necessary precautions, before presenting the AED, the simulation scenario with the camera, speakers, and microphones, and describe the function of each piece of equipment. If there is a limited supply of participants, the paramedic’s team can only be informed by the facilitators, as the main objective of this simulation is the fundamental aspects of BLSD in adults, which include immediate recognition of CA, contact with the emergency system, initiation of high-quality cardiopulmonary resuscitation, and use of AED as soon as possible.		
8 - Phase 2: Home-based and self-directed practice (Uploads). They can go back to the instructional materials for more guidance, and once satisfied with their performance, they video-record a test trial and upload it to the LMS for feedback.				8 - Phase 2: Home-based and self-directed practice (Uploads). They can go back to the instructional materials for more guidance, and once satisfied with their performance, they video-record a test trial and upload it to the LMS for feedback.		
9 - Phase 3: Feedback (How) Peers/Expert (Who) - Checklist:			Notes: 1 - Feedback can be written, checklist and video (preferably this one) so that participants can see the correct way to do it next time. In addition, these videos must be offline so that the participant can watch them several times. Alpha comments: “Here it would also be interesting to put audio and videos, mainly video with the feedback explaining where it went wrong and how the participant can correct it.” Delta comments: “Adding video explaining how to do things correctly would be very good.”	9 - Phase 3: Feedback (How) Peers/Expert (Who) - Checklist: They can go back to the instructional materials for further guidance and, once satisfied with their performance, record a video or voice and/or and send it to the LMS for feedback.		
Expected action	Findings/Justification	Completed (Y/N)		Expected action	Findings/Justification	Completed (Y/N)
1. Take the phone call and identify yourself as a healthcare professional.	A woman is crying and asking for help: The participant must collect information from the woman on the phone about the victim and the scene.	Y ( ) N ( )	No suggestions.	1. Take the phone call and identify yourself as a healthcare professional.	A woman is crying and asking for help: The participant must collect information from the woman on the phone about the victim and the scene.	Y ( ) N ( )
2. Guidance about safety.	If there are risks to the lay rescuer by scene or unsafe environments: If possible and without offering greater risks to the lay rescuer, remove the patient to a safer place as soon as possible.	Y ( ) N ( )		2. Guidance about safety.	If there are risks to the lay rescuer by scene or unsafe environments: If possible and without offering greater risks to the lay rescuer, remove the patient to a safer place as soon as possible.	Y ( ) N ( )
3. Guidance on assessing the victim's responsiveness and breathing.	The victim does not respond: The victim does not respond and help should be sent immediately to the scene.	Y ( ) N ( )		3. Guidance on assessing the victim's responsiveness and breathing.	The victim does not respond: The victim does not respond and help should be sent immediately to the scene.	Y ( ) N ( )
4. Sending help!	The participant requests that the drone and ambulance be sent: The participant quickly identified a cardiac arrest and immediately sent help.	Y ( ) N ( )		4. Sending help!	The participant requests that the drone and ambulance be sent: The participant quickly identified a cardiac arrest and immediately sent help.	Y ( ) N ( )
5. Guide to checking breathing and pulse: Guidance on checking the pulse and starting cardiopulmonary resuscitation if the pulse is absent or if he or she is in doubt.	The lay rescuer does not know if there is a central pulse: The victim still does not respond to any stimulus.	Y ( ) N ( )		5. Guide to checking breathing and pulse: Guidance on checking the pulse and starting cardiopulmonary resuscitation if the pulse is absent or if he or she is in doubt.	The lay rescuer does not know if there is a central pulse: The victim still does not respond to any stimulus.	Y ( ) N ( )
6. Guide to start cycles of 30 compressions and two ventilations: Guide the completion of three cycles of continuous compressions (200) with passive oxygen ventilation, in cases of cardiac arrest witnessed with VF/VT rhythm. I: Guide about the positioning of the hand: Rock the heel of the hand off the chest, keeping fingertips on the chest wall to maintain hand position. II: Chest compressions must have a minimum depth of 5 cm, without exceeding 6 cm. III: The delay of compressions between shock delivery must be as short as possible. IV: Resume chest compressions immediately after defibrillation for adults in cardiac arrest.	Wait for AED guidance: 1) “analyzing the heart rate”; or 2) “do not touch the patient”; and/or 3) “recommended shock, charging, move away from the patient”.	Y ( ) N ( )		6. Guide to start cycles of 30 compressions and two ventilations: Guide the completion of three cycles of continuous compressions (200) with passive oxygen ventilation, in cases of cardiac arrest witnessed with VF/VT rhythm. I: Guide about the positioning of the hand: Rock the heel of the hand off the chest, keeping fingertips on the chest wall to maintain hand position. II: Chest compressions must have a minimum depth of 5 cm, without exceeding 6 cm. III: The delay of compressions between shock delivery must be as short as possible. IV: Resume chest compressions immediately after defibrillation for adults in cardiac arrest.	Wait for AED guidance: 1) “analyzing the heart rate”; or 2) “do not touch the patient”; and/or 3) “recommended shock, charging, move away from the patient”.	Y ( ) N ( )
7. Performing 30 compressions and two ventilations, for adults in cardiac arrest. I: It is advisable to perform compressions at a frequency of 100 to 120 compressions/minute. II: Chest compressions must have a minimum depth of 5 cm, without exceeding 6 cm. III: The delay of compressions between shock delivery must be as short as possible. IV: Resume chest compressions immediately after defibrillation for adults in cardiorespiratory arrest.	Wait for AED guidance: 1) “analyzing the heart rate”; or 2) “do not touch the patient”; and/or 3) “recommended shock, charging, move away from the patient”.	Y ( ) N ( )		7. Performing 30 compressions and two ventilations, for adults in cardiac arrest. I: It is advisable to perform compressions at a frequency of 100 to 120 compressions/minute. II: Chest compressions must have a minimum depth of 5 cm, without exceeding 6 cm. III: The delay of compressions between shock delivery must be as short as possible. IV: Resume chest compressions immediately after defibrillation for adults in cardiorespiratory arrest.	Wait for AED guidance: 1) “analyzing the heart rate”; or 2) “do not touch the patient”; and/or 3) “recommended shock, charging, move away from the patient”.	Y ( ) N ( )
8. Guide to maintain the cardiopulmonary resuscitation maneuvers and the AEDs analysis until the paramedic team arrives at the scene and takes over or until the victim shows signs of life.	The paramedic team has arrived or not at the scene and will take over the case: The participant must instruct the lay rescuer to let the paramedic team take over the scenario.	Y ( ) N ( )		8. Guide to maintain the cardiopulmonary resuscitation maneuvers and the AEDs analysis until the paramedic team arrives at the scene and takes over or until the victim shows signs of life.	The paramedic team has arrived or not at the scene and will take over the case: The participant must instruct the lay rescuer to let the paramedic team take over the scenario.	Y ( ) N ( )
9. Guide to maintain the cardiopulmonary resuscitation maneuvers and the AEDs analysis until the paramedic team arrives at the scene and takes over or until the victim shows signs of life.	The victim woke up and started to move. The participant must instruct the layperson to stop the cardiac compressions, disconnect from AED machine, and wait for the paramedic's team to arrive at the location.	Y ( ) N ( )		9. Guide to maintain the cardiopulmonary resuscitation maneuvers and the AEDs analysis until the paramedic team arrives at the scene and takes over or until the victim shows signs of life.	The victim woke up and started to move. The participant must instruct the layperson to stop the cardiac compressions, disconnect from AED machine, and wait for the paramedic's team to arrive at the location.	Y ( ) N ( )
10. The participant must now instruct the woman that her father will be taken to a hospital by the paramedic team.	The paramedic team reports that the 55-year-old man returned with a central pulse and is demonstrating spontaneous breathing.	Y ( ) N ( )		10. The participant must now instruct the woman that her father will be taken to a hospital by the paramedic team.	The paramedic team reports that the 55-year-old man returned with a central pulse and is demonstrating spontaneous breathing.	Y ( ) N ( )
10 - Phase 3: Feedback (How), Peers/Expert (Who) - Free form: The feedback can be delivered in free form by providing comments.			Notes: 1 - feedback can be via checklist, written text, but also via videos explaining where you went wrong and where you got it right. This would make the participant understand exactly how he can improve his or her performance.	10 - Phase 3: Feedback (How), Peers/Expert (Who) - Upload video: Peers or experts can record videos teaching how to perform a behavior correctly and help the participant to understand through this video where they went wrong and how to improve. This video must be available offline to the participant.		
				11 - Phase 3: Feedback (How), Peers/Expert (Who) - Free form: The feedback can be delivered in free form by providing comments.		

Appendix C 

**Table 4:** The Final Version of The maxSIMdrone Training Program. AED: automatic external defibrillator BLSD: basic life support and defibrillation CA: cardiac arrest CPR: cardiopulmonary resuscitation LMS: learning management system PHOENIX: drone VF/VT: ventricular fibrillation/ventricular tachycardia 911: emergency service Y: yes N: no

Final maxSIMdrone Version		
Phase 1: Proficiency-based development of actions representations		
1 - Phase 1: Proficiency-based development of action representations (Instruction). Learning objectives: There are five learning objectives that participants are expected to learn in this course. In addition, the material must also be accessed offline. 1 - Guidance about safety ethical issues: By the end of this training program, participants should be able to communicate with rescue teams to ensure the safety of the victim and the rescuer via radio, call cellphone, or WhatsApp app. Additionally, they should be able to use acronyms for sensitive words and learn how to preserve victims' personal information to avoid further harm. Participants should be able to determine whether the location is safe for the rescuer and the victim and proceed with the care of the patient only if the location is safe. 2 - Guide assessing the victim's responsiveness: The participants should guide how the victim's responsiveness should be assessed by calling out and shaking their shoulders. If the victim responds, participants should guide the rescuer to talk to the victim and ask if he or she needs help. If the victim does not respond, immediately send the drone and a helicopter. 3 - Send help (drone and helicopter): In an extra-hospital environment, participants must immediately send the PHOENIX with an AED and a helicopter with a team of paramedics. At the same time, participants must advise on resuscitation maneuvers. It is important to designate people to be responsible for performing these functions. 4 - Guide to checking breathing and pulse: If the victim does not breathe or gasps and the pulse is absent, participants should promptly initiate cardiopulmonary resuscitation. 5 - Guide start cycles of 30 compressions and two ventilations: Participants should guide the start cycle of 30 compressions and two ventilations, considering that there is a barrier device (for example, a pocket mask to apply the ventilation).		
2 - Phase 1: Proficiency-based development of action representations (Instruction). Video demonstration of PHOENIX delivering AED and provider receiving instruction over the phone. The videos that must be in the instruction phase must contain an instructor explaining in detail each step-by-step of the training, as well as how to operate an AED and the entire BLSD in the lifesaving process. VIDEO		
3 - Phase 1: Proficiency-based development of action representations (Instruction). The lifesaving process Steps (Picture). The lifesaving process is a series of steps that can be taken to improve the chances of survival for someone experiencing sudden cardiac arrest. The steps in the lifesaving process are: 1 - Recognition and activation of the emergency response system: The first step in the lifesaving process is to recognize that someone is experiencing sudden cardiac arrest and to activate the emergency response system by calling 911. This should be done as soon as possible, as time is of the essence in these situations. 2 - Call 911: Before starting CPR, the witness should call 911, ask for help, and when instructed he or she should start the intervention. 3 - The healthcare professional sends help and provides guidance: The healthcare professional who receives the call from the witness must immediately give instructions on how to identify if the victim is breathing and if he or she has an arterial pulse. If the healthcare professional believes that the case is a cardiac arrest, he or she should immediately request the dispatch of a drone with an AED and a team of paramedics by helicopter when available. 4 - Start high-quality CPR: Initiate high-quality CPR as instructed by the healthcare professional until the AED delivered by drone or helicopter arrives. This involves chest compressions and rescue breaths to keep oxygen-rich blood circulating to the brain and other vital organs. Anyone who is trained in CPR should be able to provide this life-saving intervention. 5 - Rapid Defibrillation: The fifth step in the lifesaving process is to use a defibrillator, or AED, to deliver a shock to the heart. This can help to restore a normal heart rhythm and improve the chances of survival for someone experiencing sudden cardiac arrest. AEDs will be delivered by a drone. 6 - Advanced Life Support: The sixth step in the lifesaving process is to provide advanced life support, such as medications and other interventions, to help restore normal heart function and stabilize the person’s condition. This is typically done by paramedics or other medical professionals who are trained in advanced life support techniques. 7 - Post Arrest Care: The final step in the lifesaving process is to provide post-arrest care, such as monitoring and support for the person’s heart and other vital functions, to help them recover from the cardiac arrest. This may involve hospitalization and specialized treatment, such as coronary angiography or coronary artery bypass surgery. By following the steps in the lifesaving process, it is possible to improve the chances of survival for someone experiencing sudden cardiac arrest. It is important to act quickly and provide the appropriate interventions to help save a life.		
4 - Phase 1: Proficiency-based development of action representations (Instruction). Recommended Equipment: 1 - PHOENIX: This equipment will be used to simulate the drone controller in real time. 2 - One AED: This equipment will be used to simulate cardiac arrest treatment in which the patient would be presenting ventricular tachycardia or ventricular fibrillation. 3 - A mannequin with clothes: This simulator will be used to simulate a cardiac arrest victim. 4 - Two radios or cellphones: This equipment will be used for remote communication between the provider and the healthcare professional. 5 - Rescue team simulating that it arrived in a helicopter. Photo: Cellphones, tablets, AED, radio, drone, and stages in the lifesaving process.		
5 - Phase 1: Proficiency-based development of action representations (Assessments). In addition to the marking questionnaire, there must be the option to download the questions in a recorded voice, have offline access to these questions, and also the participant must be able to answer it by voice. To move on to the next phase, the learners must mark the action option as "very important" for all options. 1 - Take the phone call and identify yourself as a healthcare professional. Not important ( ) Important ( ) Very important ( ) 2 - Guidance about safety. Not important ( ) Important ( ) Very important ( ) 3 - Guidance on assessing the victim's responsiveness and breathing. Not important ( ) Important ( ) Very important ( ) 4 - Sending help! Not important ( ) Important ( ) Very important ( ) 5 - Guide to checking breathing and pulse: Guidance on checking the pulse and starting cardiopulmonary resuscitation if the pulse is absent or if he or she is in doubt. Not important ( ) Important ( ) Very important ( ) 6 - Guide to start cycles of 30 compressions and two ventilations: Guide the completion of three cycles of continuous compressions (200) with passive oxygen ventilation, in cases of cardiac arrest witnessed with VF/VT rhythm. I: Guide about the positioning of the hand: Rock the heel of the hand off the chest, keeping fingertips on the chest wall to maintain hand position. II: Chest compressions must have a minimum depth of 5 cm, without exceeding 6 cm. III: The delay of compressions between shock delivery must be as short as possible. IV: Resume chest compressions immediately after defibrillation for adults in cardiac arrest. Not important ( ) Important ( ) Very important ( ) 7 - Performing 30 compressions and two ventilations, for adults in cardiac arrest. I: It is advisable to perform compressions at a frequency of 100 to 120 compressions/minute. II: Chest compressions must have a minimum depth of 5 cm, without exceeding 6 cm. III: The delay of compressions between shock delivery must be as short as possible. IV: Resume chest compressions immediately after defibrillation for adults in cardiorespiratory arrest. Not important ( ) Important ( ) Very important ( ) 8 - Guide to maintaining the cardiopulmonary resuscitation maneuvers and the AEDs analysis until the paramedic team arrives at the scene and takes over or until the victim shows signs of life. Not important ( ) Important ( ) Very important ( ) 9 - Guide to maintain the cardiopulmonary resuscitation maneuvers and the AEDs analysis until the paramedic team arrives at the scene and takes over or until the victim shows signs of life. Not important ( ) Important ( ) Very important ( ) 10 - The participant must now instruct the woman that her father will be taken to a hospital by the paramedic team. Not important ( ) Important ( ) Very important ( )		
Phase 2: Home-based and self-directed practice		
6 - Phase 2: Home-based and self-directed practice (Hands-on practice). Once proficient action representations have been formed, the learners move to Phase 2 - Here, they would practice BLSD using the equipment specifically designed for this purpose and the lifesaving process steps. Photo: Cellphones, tablets, AED, radio, drone, and stages in the lifesaving process.		
7 - Phase 2: Home-based and self-directed practice (Hands-on practice). Inputs - Equipment: This simulation can be performed in a controlled environment using a mannequin, one iPad to follow video instructions, radios or cellphones for communication, and a PHOENIX and AED. This simulation is designed for both laypeople and health professionals who are part of an urgent or emergency network, such as 911, where a trained healthcare professional can assist and guide patients and victims over the phone or radio. The simulation will take place in rural or remote locations where a drone can reach the victim first, but a helicopter may be delayed. Information: This training is designed for both healthcare professionals and laypeople who work in emergency care services such as 911 in both urban and rural areas. It is also suitable for those who wish to improve their knowledge and skills in treating patients with cardiac arrest. Since the simulation takes place in a prehospital environment, there is no access to imaging or laboratory investigations. Facilitators: Two health professionals, both comfortable in providing care for patients with CA, should act as facilitators. These professionals must have experience with the use of AEDs. One should be designated as the primary facilitator, guiding participants and helping with the overall organization and execution of the case, while the second facilitator will be present to assess individual performance. Facilitators are responsible for providing participants with the appropriate information as requested. Facilitators should examine the scenario in advance to identify possible limitations or technical problems related to the proper functioning of the mannequins, radios, cellphones, iPad, PHOENIX, and AEDs. Before training begins, facilitators should explain the importance of not identifying the victim while training is taking place. This must be reinforced at all times during the program. Context: Participants initiate the case in a 911 urgent and emergency care service, which contains a healthcare professional and a licensed drone pilot (the latter can be interpreted by the facilitator). The phone rings by call or WhatsApp or a call from a radio is initiated and a 25-year-old lay-woman in CA assistant and she is desperate and crying a lot on the call. She is camping with her 55-year-old father and reports that three minutes ago he screamed with a pain in his chest and simply fell to the ground. She believes that he is not breathing. If it is by radio, sometimes the communication may fail and a third person will have the role of being a communication bridge between the woman and the health professional because this person gets a good signal from both. Prebriefing: Before starting the simulation, participants must be submitted to the fiction contract, which recognizes that everything taking place during the simulation must be treated as if they were "real" so that the objectives of the simulation can be achieved. During this period, the facilitators present the simulation scenario and all the necessary precautions, before presenting the AED, the simulation scenario with the camera, speakers, and microphones, and describe the function of each piece of equipment. If there is a limited supply of participants, the paramedic’s team can only be informed by the facilitators, as the main objective of this simulation is the fundamental aspects of BLSD in adults, which include immediate recognition of CA, contact with the emergency system, initiation of high-quality cardiopulmonary resuscitation, and use of AED as soon as possible.		
8 - Phase 2: Home-based and self-directed practice (Uploads).		
9 - Phase 3: Feedback (How), Peers/Expert (Who) - Checklist: They can go back to the instructional materials for further guidance and, once satisfied with their performance, record a video or voice and/or send it to the LMS for feedback.		
Expected action	Findings/Justification	Completed (Y/N)
1. Take the phone call and identify yourself as a healthcare professional.	A woman is crying and asking for help: The participant must collect information from the woman on the phone about the victim and the scene.	Y ( ) N ( )
2. Guidance about safety.	If there are risks to the lay rescuer by scene or unsafe environments: If possible and without offering greater risks to the lay rescuer, remove the patient to a safer place as soon as possible.	Y ( ) N ( )
3. Guidance on assessing the victim's responsiveness and breathing.	The victim does not respond: The victim does not respond and help should be sent immediately to the scene.	Y ( ) N ( )
4. Sending help!	The participant requests that the drone and ambulance be sent: The participant quickly identified a cardiac arrest and immediately sent help.	Y ( ) N ( )
5. Guide to checking breathing and pulse: Guidance on checking the pulse and starting cardiopulmonary resuscitation if the pulse is absent or if he or she is in doubt.	The lay rescuer does not know if there is a central pulse: The victim still does not respond to any stimulus.	Y ( ) N ( )
6. Guide to start cycles of 30 compressions and two ventilations: Guide the completion of three cycles of continuous compressions (200) with passive oxygen ventilation, in cases of cardiac arrest witnessed with VF/VT rhythm. I: Guide about the positioning of the hand: Rock the heel of the hand off the chest, keeping fingertips on the chest wall to maintain hand position. II: Chest compressions must have a minimum depth of 5 cm, without exceeding 6 cm. III: The delay of compressions between shock delivery must be as short as possible. IV: Resume chest compressions immediately after defibrillation for adults in cardiac arrest.	Wait for AED guidance: 1) “analyzing the heart rate”; or 2) “do not touch the patient”; and/or 3) “recommended shock, charging, move away from the patient”.	Y ( ) N ( )
7. Performing 30 compressions and two ventilations, for adults in cardiac arrest. I: It is advisable to perform compressions at a frequency of 100 to 120 compressions/minute. II: Chest compressions must have a minimum depth of 5 cm, without exceeding 6 cm. III: The delay of compressions between shock delivery must be as short as possible. IV: Resume chest compressions immediately after defibrillation for adults in cardiorespiratory arrest.	Wait for AED guidance: 1) “analyzing the heart rate”; or 2) “do not touch the patient”; and/or 3) “recommended shock, charging, move away from the patient”.	Y ( ) N ( )
8. Guide to maintain the cardiopulmonary resuscitation maneuvers and the AEDs analysis until the paramedic team arrives at the scene and takes over or until the victim shows signs of life.	The paramedic team has arrived or not at the scene and will take over the case: The participant must instruct the lay rescuer to let the paramedic team take over the scenario.	Y ( ) N ( )
9. Guide to maintain the cardiopulmonary resuscitation maneuvers and the AEDs analysis until the paramedic team arrives at the scene and takes over or until the victim shows signs of life.	The victim woke up and started to move. The participant must instruct the layperson to stop the cardiac compressions, disconnect from AED machine, and wait for the paramedic's team to arrive at the location.	Y ( ) N ( )
10. The participant must now instruct the woman that her father will be taken to a hospital by the paramedic team.	The paramedic team reports that the 55-year-old man returned with a central pulse and is demonstrating spontaneous breathing.	Y ( ) N ( )
10 - Phase 3: Feedback (How), Peers/Expert (Who) - Upload and download video. Peers or experts can record videos teaching how to perform a behavior correctly and help the participant to understand through this video where they went wrong and how to improve. This video must be available offline to the participant.		
11 - Phase 3: Feedback (How), Peers/Expert (Who) - Free form: The feedback can be delivered in free form by providing comments.		
